# Efficacy of COMPAs, an App Designed to Support Communication Between Persons Living With Dementia in Long-Term Care Settings and Their Caregivers: Mixed Methods Implementation Study

**DOI:** 10.2196/47565

**Published:** 2024-07-04

**Authors:** Ana Inés Ansaldo, Michèle Masson-Trottier, Barbara Delacourt, Jade Dubuc, Catherine Dubé

**Affiliations:** 1 Laboratoire de Plasticité cérébrale, Communication et Vieillissement Centre de recherche de l'Institut Universitaire de gériatrie de Montréal Université de Montréal Montréal, QC Canada; 2 John Hopkins Hospital John Hopkins University Baltimore, MD United States

**Keywords:** dementia, communication, caregivers, technology, burden, mixed methods design, quality of life, mobile phone, tablet

## Abstract

**Background:**

Persons living with dementia experience autonomy loss and require caregiver support on a daily basis. Dementia involves a gradual decline in communication skills, leading to fewer interactions and isolation for both people living with dementia and their caregivers, negatively impacting the quality of life for both members of the dyad. The resulting stress and burden on caregivers make them particularly susceptible to burnout.

**Objective:**

This study aims to examine the efficacy of Communication Proches Aidants (COMPAs), an app designed following the principles of person-centered and emotional communication, which is intended to improve well-being in persons living with dementia and caregivers and reduce caregiver burden.

**Methods:**

In this implementation study, volunteer caregivers in 2 long-term care facilities (n=17) were trained in using COMPAs and strategies to improve communication with persons living with dementia. Qualitative and quantitative analyses, semistructured interviews, and questionnaires were completed before and after 8 weeks of intervention with COMPAs.

**Results:**

Semistructured interviews revealed that all caregivers perceived a positive impact following COMPAs interventions, namely, improved quality of communication and quality of life among persons living with dementia and caregivers. Improved quality of life was also supported by a statistically significant reduction in the General Health Questionnaire-12 scores (caregivers who improved: 9/17, 53%; *z*=2.537; *P=*.01). COMPAs interventions were also associated with a statistically significant increased feeling of personal accomplishment (caregivers improved: 11/17, 65%; *t*_15_=2.430; *P=*.03; *d*=0.61 [medium effect size]).

**Conclusions:**

COMPAs intervention improved well-being in persons living with dementia and their caregivers by developing person-centered communication within the dyad, increasing empathy, and reducing burden in caregivers although most caregivers were unfamiliar with technology. The results hold promise for COMPAs interventions in long-term care settings. Larger group-controlled studies with different populations, in different contexts, and at different stages of dementia will provide a clearer picture of the benefits of COMPAs interventions.

## Introduction

### Background

Dementia is a progressive or chronic syndrome, affecting memory, reasoning, orientation, understanding, calculation, learning ability, language, and judgment; it represents a greater impairment of cognitive function than might be expected, while being the main cause of disability and dependence among older people [1]. Dementia is the consequence of diverse diseases, the most common being Alzheimer disease [1].

According to the World Health Organization, 55 million individuals worldwide live with dementia, making it one of the main causes of disability and social deprivation among older adults [2]. These figures are set to rise over the next few years, with an estimated 78 million people expected to be living with dementia by 2030. The report states that support for the care of people living with dementia and help for caregivers need to be stepped up urgently. As dementia progresses, it has an impact on the autonomy of the person affected. At the advanced stage, persons living with dementia can no longer live at home because they are no longer able to carry out everyday tasks (eg, dressing, eating, and washing), and this is often difficult to manage for those around them.

### Person-Centered Communication and Dementia

Dementia is characterized by a progressive deterioration of cognitive functions and particularly affects language [3]. As the disease progresses, persons living with dementia will experience increasing communication deficits that impact all aspects of life. Frequent communication breakdowns lead to feelings of frustration that can trigger reactive behaviors known as behavioral and psychological symptoms of dementia (BPSD) [4]. Communication breakdowns complexify care, increase caregiver burden, and decrease the quality of life (QoL) for persons living with dementia and caregivers [5,6]. In the later stages of dementia, sustaining a simple communicative exchange (eg, greetings or short conversation between the caregiver and the person living with dementia) is practically impossible. Because they are unable to express their needs or understand others, persons living with dementia often express frustration [7] and generally require more attention than other older adults, which also contributes to increasing the burden on caregivers and decreasing QoL [8,9].

The literature shows that communication is a key component of quality care [10-13] and a core component of person-centered care, promoting positive social interactions around topics of the life story of persons living with dementia [5]. Person-centered care, considered state of the art in dementia care [14], recognizes the individual as a person and aims to respond to the individual’s feelings, preferences, and needs [15,16]. Furthermore, person-centered care precludes perceiving the person living with dementia exclusively as a person with illness, and such perception contributes to cognitive decline, adds to communication difficulties, and contributes to depersonalization [13]. A person-centered nondirective approach considers the person’s lifestyle, culture, and history, including their likes and dislikes, preferences, and interests, while always considering the person’s point of view [5].

In sum, communication breakdowns contribute to depersonalization and weakening of person-centered care [17], requiring continuous adaptation from the caregiver. In contrast, person-centered care develops leadership in caregivers, prompting management changes toward a more personalized philosophy of care.

### Impact of Communication on Care

Communication deficits in the context of dementia have negative impacts on several aspects of the caregiver and resident relation within a long-term care (LTC) setting. More specifically, the progressive nature of the illness entails frequent communication breakdowns, which generate frustration in both the caregiver and the person living with dementia (referred to as the dyad) [5,6]. Indeed, most persons living with dementia show signs of frustration when they cannot understand a conversation or make themselves understood [7,18]. Frustration increases emotional tension, which in turn contributes to the caregiver’s burden [9]. Poor-quality interactions also increase the risk of agitation and apathy in persons living with dementia [8]. While the quality of communication within the dyad is known to modulate caregiver burden [9], it also affects person-centered care, both of which are essential to QoL [19,20]. Greater burden and a higher prevalence of anxiety disorders are observed in caregivers working with persons living with dementia [21,22]. Burden is described along 2 dimensions: objective burden, which refers to the degree of dependence of the person living with dementia and the presence of BPSD, and subjective burden, which is associated with the physical, social, and emotional dimensions of caring, as well as the resources available to the caregiver [23]. A systematic review by Queluz et al [24] grouped professional caregiver needs into 3 main themes: emotional health, formal or informal help received from third parties, and need for information about dementia and associated care.

Several variables related to the physical and social environment in which communication occurs can create living conditions that promote or hamper QoL in caregivers and persons living with dementia [20]. These include the quality of caregivers’ engagement in care (ie, a positive attitude), enjoyable communication as reflected by personalized exchanges [25], and social activities [26]. In addition, the progression in communication deficits often results in avoidance of communication within the dyad, a factor that contributes to accelerating cognitive decline and triggering BPSD, which are particularly disruptive in LTC settings [27]; it also impacts caregivers’ QoL [28]. A review by Scott-Cawiezell et al [29] on 995 staff members has shown that improvements are required to achieve open, accurate, and timely communication in nursing homes. More specifically, according to McCormack et al [30], while some staff members know of residents’ preferences, this information is not routinely communicated to all staff members in a facility. By sharing information about communication topics and strategies facilitating person-centered communication with each resident, caregivers may become more efficient in providing care and less exposed to communication breakdowns and the resulting increase in their burden. Moreover, according to Kolanowski et al [31], available communication systems do not consider the time and resource constraints of nursing homes.

There is a strong consensus on the need to empower caregivers and give them strategies so that they can optimize communication with persons living with dementia [28]. Particularly in the context of LTC residences, the evidence shows that adopting a person-centered care approach significantly influences quality of care and the QoL of both caregivers and persons living with dementia, by improving care compliance and reducing caregivers’ burden [26,28]. Moreover, the literature shows that personalized, emotionally relevant contents facilitate person-centered care, preserve personhood, and prevent dehumanization and isolation of persons living with dementia [5]. Sharing complete information with the persons living with dementia and their families, ensuring participation of persons living with dementia and their families in decision-making, and securing the collaboration of persons living with dementia in policy and program development are among the key elements of person-centered care [32]. Thus, while communicating with persons living with dementia in the provision of care, it is important to consider the unique life history, feelings, cultural background, values, and preferences of each person. This consideration is a challenge for a caregiver in an LTC setting who meets a person living with dementia in the advanced stages when they are unable to tell their life story. Communication tools that consider this important aspect of person-centered care while fitting into the reality of LTC settings (including tight schedules, resources, and constraints, together with administrative investment in nursing leadership) are therefore required to achieve beneficial changes.

Sustaining person-centered care and communication between persons living with dementia and their caregivers, especially in LTC settings, requires adapted communication tools. It was with this purpose that our team designed Communication Proches Aidants (COMPAs; it also refers to the compass, the instrument that orients sailors in troubled seas).

COMPAs was designed to support person-centered communication between persons with severe communication impairments and their caregivers, professional or informal. It is based on the concept of person-centered communication, as reflected by its personalized audiovisual content in line with the life trajectory, preferences, interests, and culture of the persons living with dementia. Through coviewing, it engages the person living with dementia and the caregiver in a form of dialogue beyond words as they share verbal and nonverbal expressions of joy and well-being. Unlike the purely transactional communication that is characteristic of basic care [17], COMPAs puts the person living with dementia at the center of communication, thereby providing a unique framework for person-centered care in the context of dementia.

In sum, the person-centered care approach acknowledges the person living with dementia as their own person. Communication is an essential tool in the implementation of this approach and the maintenance of personhood [33]. Persons living with dementia have trouble expressing their needs, which often leads to the perception that they have no awareness, and their interactions lack mutuality [33]. However, persons living with dementia need meaningful interactions [34], and caregivers need adequate communication tools to meet the care and social needs of persons living with dementia. Unfortunately, caregivers lack sufficient communication training and adapted tools to support social communication in order to overcome the communication deficits of persons living with dementia. Furthermore, LTC settings are environments where there are multiple, often changing, caregivers who revolve around the persons living with dementia. Technology could play a crucial role in care for persons living with dementia. According to Koo and Vizer [35], technology facilitates daily activities, maintains social interactions, supports autobiographical memory, and promotes leisure activities, all while allowing storing and monitoring the clinical status of individuals. Overall, technology could improve the QoL of persons living with dementia, reducing the BPSD and burden on caregivers [35].

### Technology and Communication

#### Overview

Although the evidence shows that technology is relevant for promoting social interactions [35-37], there is a need for rigorous studies on the use of digital tablets in the context of persons living with dementia and their caregivers. Specifically, a scoping review on this topic points to the benefits of technology for intergenerational communication [38], in particular, by means of suggested conversation topics related to the life trajectory of the person living with dementia, which generate positive emotions; however, the review acknowledged that little attention is paid to higher-level needs, such as self-esteem and preserving personhood [35]. Regarding the use of tablets, evidence shows that persons living with dementia may enjoy using tablets at all stages of dementia [38,39]. Hung et al [40] has pointed out the utility of tablets in the context of one-on-one, small-group, and large-group activities, thereby facilitating relationship building and resident engagement and helping caregivers gain better knowledge of the interests and abilities of the persons living with dementia. Furthermore, a recent review [37] has identified a series of apps that could potentially prevent and overcome communication barriers. However, none of these apps were designed to promote person-centered communication or were tested in an LTC setting. Finally, a recent scoping review [35] on the use of technology in LTC has shown some positive impacts on behavior engagement and mood in LTC residents. Importantly, the authors highlight that this finding is not specific to persons living with dementia, who are generally excluded from such studies, while pointing out the importance of examining the impact of technology use specifically in this population [35].

In sum, technology offers some promise for supporting communication in persons with dementia. However, there is limited empirical research on the use of technology to support communication between caregivers and residents in LTC residences [35,41], and only 1 study examined the caregivers’ perspectives on the ability of mobile apps to support caregiver-resident communication [36]. The consensus in the literature is that more research on the use of communication apps involving persons living with dementia in LTC settings is imperative [35]. Moreover, there is a need for evidence-based apps specifically designed to promote person-centered communication in cases of advanced dementia when the possibilities of verbal communication are very limited.

#### COMPAs App

COMPAs is an evidence-based app available on digital tablets to facilitate portability and participation of persons living with dementia. It is designed to support person-centered care between persons living with dementia and their caregivers. This app integrates knowledge from proven therapies [6,15,42-46] and has the advantage of combining these concepts in a single medium [47]. COMPAs is a secure platform that collects photos, music, and videos that have marked the life of the persons living with dementia. It was designed by speech language pathologists (SLPs) and media experts and offers an intuitive environment to promote positive communicative moments; its content is fully customizable to reflect the relevant life events of each person living with dementia and is constantly adaptable as the person’s dementia evolves.

With COMPAs, caregivers gain secure access to personalized libraries of audiovisual materials that are selected according to residents’ personal preferences, cultural background, and life history. Specifically, photographs, music, and videos can be uploaded to a secure space through the tablet or the COMPAs website, allowing families to remotely add content to enrich their loved one’s COMPAs space. Apart from the content provided by the family, the COMPAs artificial intelligence module provides access to personalized internet content related to the person’s life history (eg, places where the person lived, favorite sports, animals, and hobbies). Caregivers can also add music pieces (from music libraries available on the tablet or through a Spotify account) as well as excerpts from movies or shows available on YouTube, while family caregivers can safely add personal videos. A “like” allows users to highlight particularly meaningful content. Before closing COMPAs each time, the caregiver is given a short multiple-choice questionnaire, which gathers a more personal perspective on the COMPAs session, while providing a means to follow up its effects and share relevant information on session outcomes with the members of the team. Coviewing sessions with COMPAs have been shown to facilitate person-centered care, the gold standard in dementia care. Studies conducted during the COVID-19 pandemic showed that COMPAs sessions triggered emotional communication, characterized by shared verbal and nonverbal exchanges related to positive emotions, while increasing social engagement between persons living with dementia and caregivers [47]. Finally, by adding likes to specific music, videos, or photographs in the residents’ personalized space and writing comments, caregivers can share information with the team about the best communication strategies and topics to sustain person-centered communication.

**Figure 1:**
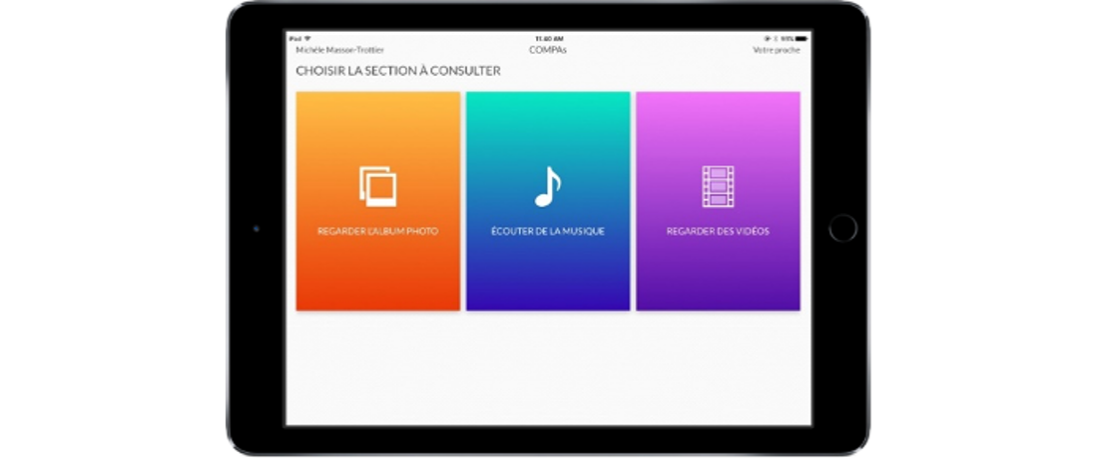
Communication Proches Aidants (COMPAs) interface.

The application was initially developed in French; however, because it uses little written language and is very intuitive, neither language impairments nor language barriers prevent its use by speakers of other languages (see Figure 1 showing the app’s interface).

The rationale behind COMPAs lies in the person-centered care approach and emotional communication. Its personalized, meaningful content reduces the impact of communication deficits by encouraging nonverbal and emotional communication. Regulation of positive emotions has been shown to optimize care in the context of dementia, while favoring trust and promoting well-being for the person living with dementia [48] and the caregiver [49].

Pilot work by our team has shown that COMPAs allows caregivers and LTC residents to enjoy moments of person-centered interaction, breaking the vicious circle of noncommunication, thereby reducing both residents’ isolation and caregivers’ burden. COMPAs’s theoretical background and our pilot findings offer some promise regarding the app’s potential to support person-centered communication between persons living in LTC residences and their caregivers.

### Purpose of the Study

In light of these pilot findings and considering the need for evidence-based technology tools to support person-centered communication between persons living with dementia and their caregivers in LTC settings [35,41], the purpose of this study was to test the use of COMPAs in LTC settings. Specifically, we implemented COMPAs in the context of LTC daily routines and measured its effects on the communication between residents and caregivers, caregivers’ burden, and the QoL of persons living with dementia and caregivers.

In line with the literature, and considering the rationale underlying COMPAs, it was expected that interventions with COMPAs would achieve the following:

1. Improve the quality of communication between the person living with dementia and the caregiver, as measured through improvements in questionnaire scores and semistructured interviews.
2. Enhance the QoL of the persons living with dementia, as measured through improvements in questionnaire scores and semistructured interviews.
3. Reduce the burden on caregivers, as measured through improvements in questionnaire scores and semistructured interviews.

In addition, we anticipated that COMPAs would be adapted to the LTC environment. This had been assessed through participant adherence and satisfaction with the use of COMPAs in this study.

## Methods

### Study Design

**Figure 2:**
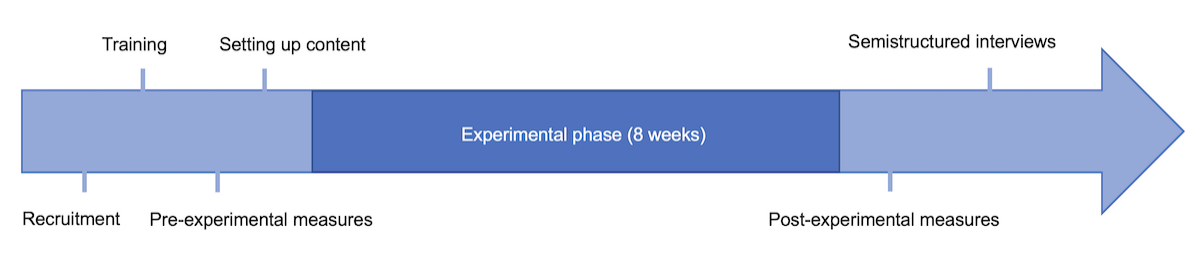
Study timeline.

This study used a pretest-posttest experimental design, with a COMPAs intervention administered over 8 weeks. The total duration of the study, including recruitment and assessments, was 14 weeks. The timeline depicted in Figure 2 shows the various stages of the COMPAs study.

### Participant Selection Process, Inclusion, and Exclusion Criteria

#### Caregivers

In total, 17 caregivers were recruited. The inclusion criteria were being a caregiver at the Paul-Bruchési LTC Center in Montreal, Québec, Canada, or Saint-Victor LTC center in Amiens, France, and caring for a person living with dementia who presented communication impairments as described in their chart or perceived by the caregiver. There were no exclusion criteria for caregivers. However, 1 participant from the Saint-Victor center was subsequently excluded from the study analyses, as only quantitative data were available for this participant; consequently, 16 caregivers completed the study. Most of the caregivers included in this study were women (14/17, 82%). Caregivers were between the ages of 24 and 57 years and had between 1 and 29 years of work experience in the health sector. Before this study, none of the caregivers had used a digital tablet, and only some used a smartphone (4/17, 22%) at work, although they were all familiar with these technologies since more than half of them used a digital tablet (10/17, 59%) or a smartphone (15/17, 88%) at home. Of the 22 caregivers who attended the information session and were not included in the study, 3 (14%) were not able to participate because they were transferred to another workplace, 1 (4%) refused to participate for personal reasons, and 1 (4%) left on parental leave.

#### Residents

The inclusion criteria were having an assigned caregiver enrolled in the project and experiencing communication difficulties, as identified by a caregiver, in the context of a diagnosis of major neurocognitive impairment, whether isolated or in combination with hearing and visual loss, or other conditions that can challenge communication, including a linguistic barrier. To make the samples as representative of the LTC population as possible, there were no exclusion criteria for persons living with dementia. A total of 17 residents participated in the study; they were aged between 61 and 96 years. Most of the residents (11/17, 65%) had a diagnosis of dementia (mixed dementia: n=6, 35%; Alzheimer disease: n=3, 18%; vascular dementia: n=1, 6%; severe dementia: n=1, 6%; and Lewy body disease: n=1, 6%). Other diagnoses were hippocampal atrophy (n=1, 6%), generalized anxiety (n=1, 6%), and cancer (n=1, 6%). Most of the residents (n=12, 71%) included in this study were women and still had some ability to express themselves verbally in isolated words or short utterances, with fluctuating comprehension of short sentences (n=15, 88%), including due to hearing limitations (n=6, 36%).

### Recruitment Process

The recruitment process for caregivers was on a voluntary basis. Specifically, the project was presented by the research assistant (RA) and the laboratory director (Ana Inés Ansaldo) during a staff meeting. The purpose of the presentation was to stimulate interest in the study and to introduce COMPAs to the staff members. Staff members were asked to contact the head of the LTC unit to express their interest in participating in the study. Each caregiver identified a resident with whom they wanted to improve communication. The research team then asked the residents if they were interested in participating.

### Ethical Considerations

Ethics approval was obtained from the Centre de recherche de l'Institut universitaire de gériatrie de Montréal (CRIUGM) Ethics Committee (approval number CER-18-19-14), and informed written consent was obtained from the residents or their representatives in cases of incapacity. Caregivers were invited to sign consent forms with the RA after they had expressed interest to participate in the study.

### Pre-Experimental Phase

#### Information Session

The first week of the study was dedicated to describing the project’s purpose, the procedures, the measurement tools, and COMPAs’s characteristics and use.

#### Training Sessions on COMPAs Use

Caregivers received two 30-minute training sessions on COMPAs during the daily planned team meetings. Facilitated by an SLP or a trained RA, the training session focused on COMPAs’s rationale and principles and a demonstration of its use by the trainer. There was a hands-on practice session at the end of the training.

#### Pre-Experimental Measures

The measures administered to caregivers included questionnaires investigating QoL (General Health Questionnaire-12 [GHQ-12] items) [50] and the burden at work (Maslach Burnout Inventory [MBI]) [51]. The RA also administered measures related to residents, including an overview of residents’ communication deficits (*Grille d’évaluation des difficultés de communication dans la démence* [GCOM]) [52] and a QoL questionnaire (*Qualité de vie dans la démence de type Alzheimer* [QDV-DTA]) [53]. Information on the residents’ and caregivers’ age, sex, and other sociodemographic data was also collected, as was information on residents’ neurocognitive disorders from their medical charts.

The GHQ-12 was used to acquire data on the caregivers’ general QoL. It includes 12 questions, scored on a 4-point Likert scale where 1=not at all, 2=not more than usual, 3=a little less than usual, and 4=a lot more than usual. In the standard scoring system, scores of 1 or 2 are given 0 point, and scores of 3 or 4 are given 1 point. The overall score is the sum of 0 and 1 points. If the sum is higher than 2, it is considered problematic.

The MBI measures caregiver burden at work. It comprises (1) an emotional exhaustion score (9 questions; scores of <17, 18-29, and >30 denote low, moderate, and high emotional exhaustion levels, respectively); (2) a depersonalization score (5 questions; scores of <5, 6-11, and >12 denote low, moderate, and high empathy loss, respectively); and (3) a personal achievement score (8 questions; scores >40, 34-39, and <33 denote low, moderate, and high achievement levels, respectively). Caregivers were asked to rate their own scores on this test.

The GCOM measures the severity of communication difficulties. The caregiver is asked to score communication behaviors for each resident (eg, “The person has word-finding difficulties”) on a Likert scale ranging from 1 (always) to 4 (never); it is also possible to select “does not speak enough for me to judge.” All scores are added up to give a final communication score. The QDV-DTA measures residents’ QoL. It consists of 13 questions, with higher scores indicating better QoL.

#### Setting Up Personalized COMPAs Libraries

Before the intervention, the RA completed a personal history with the residents’ representatives. The caregivers, along with an SLP or a trained RA, created a personalized communication space for each resident by adding significant personal content such as personal photos, images, videos, and music selected based on their life history questionnaire and the information provided by the residents’ representatives.

### Experimental Phase

Caregivers used COMPAs for 8 weeks in the context of their daily LTC routine (eg, family visits; recreation time; birthday celebrations; and situations triggering reactive behaviors, such as personal care or specific interventions). COMPAs sessions could be very short (2-5 minutes) or longer (up to 20 minutes). They consisted of coviewing sessions (ie, resident and caregiver) during which personalized material in the resident’s library was presented by the caregiver, with the purpose of eliciting positive emotions and triggering verbal and nonverbal exchanges within the dyad, according to the principles learned during the training sessions.

Caregivers were instructed to ensure that residents always used their hearing and visual aids during COMPAs sessions. Furthermore, following caregivers’ comments, we made adaptations such as using personal hearing amplifiers or Bluetooth speakers to improve listening to the music. In addition, since COMPAs is a person-centered approach, we encouraged caregivers to focus on the person’s strengths and therefore used more photos and videos with people with hearing loss and more music and audio clips with those with visual loss.

Caregivers could modify the number and duration of COMPAs sessions according to what was possible for them (eg, workload and residents’ status), as long as they respected a minimum of 15 minutes a day, at a time they considered appropriate. During the first 2 weeks of the intervention, with the purpose of facilitating COMPAs use, the SLP and the trained RA provided direct support to the caregivers on the telephone, by email, or during visits to the LTC facility. All caregiver shifts were covered so that everyone had the chance to ask questions. Indirect support (by telephone and email) was available throughout the 8-week duration of the intervention.

### Postexperimental Phase and Measures

Following the 8 weeks of COMPAs intervention, the same measures administered before the experiment were administered by the RA to the caregivers and residents. Semistructured interviews with the participating caregivers were also completed: individual, semistructured 20- to 40-minute interviews were conducted with the caregivers in person, and on the Zoom (Zoom Video Communications Inc) platform. Only the interviewer and the caregiver were present at the meeting. The questions came from an interview guide developed by the last author (CD), based on interview guides from previous studies [54,55]. The interview consisted of open-ended questions and began with a very general question (“Could you please talk about your experience with COMPAs in the last few weeks?”). Uncertainties arising from participants’ answers were elucidated with follow-up questions. At the end, the interviewer also asked the caregiver if they wished to provide any additional information. Overall, 17 interviews were conducted with the 17 participants.

### Data Analysis Plan

#### Quantitative Analyses

Data analyses include quantitative and qualitative approaches. Primary outcome measures are scores on the GHQ-12 [50] and the BMI [51] with caregivers and scores on the GCOM [52] and QDV-DTA [53] with persons living with dementia. The 4 outcome measures were used as dependent variables, and the 2 measurement points before and after the COMPAs intervention were considered as independent variables. The results were entered in paired sample 2-tailed *t* tests with an **α** of .05 to define significance. The paired-sample *t* test allows to control for individual variables that potentially affect outcome measures. Furthermore, paired-sample *t* tests are suitable to analyze data from small samples, as in this study. When the assumptions of the paired-sample *t* test were not fulfilled (ie, difference scores were not normally distributed and within-participant variability was not consistent), a nonparametric alternative, the Wilcoxon signed-rank test, was used. For all measures, outliers were detected by inspection; criteria to exclude data from analyses were set at values >1.5 box lengths from the edge of the box plot.

#### Qualitative Analyses

The qualitative measures involved a qualitative content analysis approach [56], as described by Intissar and Rabeb [57] and Vallée et al [58]. To analyze data from semistructured interviews with caregivers, MAXQDA software (VERBI GmbH), a qualitative analysis program for discourse content analyses was used [59]. The interviews were audio recorded and manually transcribed verbatim by BD and JD, and the interjudge validity was assessed, followed by thematic coding. The corpus of the interviews was read multiple times separately by 2 authors to achieve a full understanding of the data. Transcripts were coded individually by 2 authors (CD and JD), and multiple meetings took place to reach intercoder reliability. Each author separately generated codes, following which they met to discuss them and reached consensus on the coding tree, which was further discussed with the last author (CD) of this manuscript. Quotations presented in this paper were translated from French into English. Caregivers were assigned numbers from P1 to P17.

## Results

### Quantitative Findings

#### Caregivers

Outlier values were detected in the difference scores for the GHQ-12, the MBI-depersonalization score and the MBI-personal achievement score. All data points >1.5 box lengths, each associated with a different participant, were removed from this specific analysis. The differences between the MBI-emotional exhaustion score, MBI-depersonalization score, and MBI-personal achievement score scores were normally distributed (*P=*.39, *P=*.29, and *P=*.74, respectively). Thus, paired-sample *t* tests were used to analyze these differences. The results are presented in Table 1. In contrast, the GHQ-12 scores were not normally distributed (*P=*.02), and therefore, a Wilcoxon signed-rank test was used to analyze these differences. The results are presented in Table 2. Data are presented as mean (SD), unless otherwise stated.

**Table 1:** Individual scores and mean (SD) values on subsections of the Maslach Burnout Inventory.

		Emotional exhaustion				Depersonalization				Personal achievement		
		Pretest	Postest	Difference	Pretest		Posttest	Difference	Pretest		Posttest	Difference
**ID (scores)**												
	CA^a^3	43	33	–10	18		11	–6	38		36	–2
	CA4	18	21	3	9		6	–3	43		45	2
	CA6	16	19	3	6		6	0	45		46	1
	CA7	20	4	–16	0		0	0	45		48	3
	CA10	9	28	19	13		17	–2	31		33	2
	CA11	17	15	–2	7		4	–4	43		39	–4
	CA15	10	7	–3	11		6	–5	41		47	6
	CA16	14	6	–8	4		1	–1	44		44	0
	FR^b^1	14	14	0	6		14	8	37		27	–10
	FR2	11	1	–10	11		5	–7	43		42	–1
	FR4	20	22	2	15		6	–9	42		44	2
	FR5	11	12	1	1		2	2	35		39	4
	FR6	20	9	–11	5		5	0	32		33	1
	FR7	54	51	–3	6		5	–1	45		44	–1
	FR8	13	16	3	6		2	1	39		44	5
	FR9	36	33	–3	6		14	8	26		36	10
	FR10	2	15	13	4		13	12	4		11	7
**Values**												
	Group, mean (SD)	19.29 (13.2)	18.00 (12.67)	–1.29 (8.68)	7.53 (4.77)		6.88 (5.06)	–0.41 (5.57)	37.24 (10.21)		38.71 (9.20)	1.47 (4.57)
	Outlier, mean (SD)	—^c^	—	—	7.75 (4.83)		6.5 (4.96)	1.18 (4.70)	37.25 (10.54)		39.43 (8.98)	2.18 (3.60)

^a^CA: caregivers from the Canadian site.

^b^FR: caregivers from the French site.

^c^Not applicable.

**Table 2:** Individual scores for caregivers on the General Health Questionnaire-12 (GHQ-12) and pre- and postintervention mean scores with all participants.

			GHQ-12				
			Pretest		Posttest		Difference
**ID**							
	CA^a^3	4		3		–1	
	CA4	0		1		1	
	CA6	3		3		0	
	CA7	0		0		0	
	CA10	3		0		–3	
	CA11	0		0		0	
	CA15	1		0		–1	
	CA16	0		0		0	
	FR^b^1	0		0		0	
	FR2	0		0		0	
	FR4	2		0		–2	
	FR5	1		0		–1	
	FR6	5		2		–3	
	FR7	8		3		–5	
	FR8	0		0		0	
	FR9	2		1		–1	
	FR10	3		0		–3	
**Values**							
	Group, mean (SD)	1.88 (2.26)		0.76 (1.20)		–1.11 (1.57)	
	Mean without outliers (SD)	1.5 (1.67)		0.62 (1.08)		–0.87 (1.25)	

^a^CA: caregivers from the Canadian site.

^b^FR: caregivers from the French site.

To determine whether the use of COMPAs by caregivers in LTC settings influenced their scores on the GHQ-12 questionnaire, the Wilcoxon signed-rank test was used to measure QoL scores. The difference scores were approximately symmetrically distributed, as assessed by a histogram with a superimposed normal curve. Of the 17 caregivers who participated in this study, 9 (53%) showed a decrease in score difference, 7 (41%) showed a tied score, and 1 (6%) showed an increase in score difference. There was a statistically significant change (mean –0.88, SD 1.26; median –0.5, IQR 2.50) between GHQ-12 scores at T3 (mean 1.50, SD 1.67; median 1, IQR 3.0) and at T14 (mean 0.63, SD 1.09; median 0, IQR 1.5; *z*=2.54; *P=*.01). At T14, 1 (6%) caregiver’s score had worsened, 9 (53%) had improved, and 7 (41%) remained the same.

A 2-tailed paired-sample *t* test was used to determine whether COMPAs influenced caregivers’ scores on the MBI-emotional exhaustion scale, MBI-depersonalization scale, and MBI-personal achievement scale. No significant change from T3 (mean 19.29, SD 13.20) to T14 (mean 18.00, SD 12.67; t_16_=–0.62; *P=*.55) was found on the MBI-emotional exhaustion scale. Specifically, at T3, 10 (59%) caregivers scored a low level of emotional exhaustion, 4 (24%) scored a moderate level, and 3 (18%) scored an elevated level. At T14, 10 (59%) caregivers were at a low level, 4 (24%) at a moderate level, and 3 (18%) at an elevated level; scores had worsened for 7 (41%) caregivers, improved for 9 (53%), and remained the same for 1 (6%).

There was also no significant change on the MBI-depersonalization scale (T3: mean 7.75, SD 4.84; T14: mean 6.88, SD 5.06; t_15_=–1.01; *P=*.33). At T3, 5 (29%) caregivers had a low level of empathy loss, 9 (53%) had a moderate level, and 3 (18%) had a high level. At T14, 8 (47%) caregivers scored a low level of empathy loss, 5 (29%) scored a moderate level, and 4 (24%) scored a high level; 9 (53%) caregivers had improved, 5 (29%) had worsened, and 3 (18%) remained at the same level.

Regarding personal achievement, according to the MBI-personal achievement scale, there was a significant change following COMPAs use (T3: mean 37.24, SD 10.21; T14: mean 38.71, SD 9.2; t_15_=2.43; *P=*.03; *d*=0.61 for a medium effect size). Specifically, at T3, a total of 9 (53%) caregivers scored a high level of personal achievement, 4 (24%) scored a moderate level, and 4 (24%) scored a low level. At T14, 9 (53%) caregivers scored a high level of personal achievement, 4 (24%) scored a moderate level, and 4 (24%) scored a low level; 5 (29%) caregivers had worsened, 11 (65%) had improved, and 1 (6%) remained the same.

#### Residents

A paired-sample *t* test was used to determine whether there was a statistically significant mean difference between residents’ overall scores on the QDV-DTA and the GCOM before and after the use of COMPAs by caregivers in the LTC setting. There were no outliers in the data or in the QDV-DTA differences or in the GCOM differences, as assessed by the inspection of a box plot for values >1.5 box lengths from the edge of the box.

The assumption of normality was not violated, as assessed by the Shapiro-Wilk test for the QDV-DTA or the GCOM (*P=*.78 and *P=*.78, respectively). The results did not reveal any significant change in the overall score on the QDV-DTA (QDV-DTA: preintervention mean 33.42, SD 3.92; postintervention mean 34.58, SD 5.42; t_11_=0.84; *P=*.42) or the GCOM (GCOM: preintervention mean 16.42, SD 10.08; postintervention mean 20.67, SD 12.92; t_11_=1.89; *P=*.09). However, 2-tailed *t* tests revealed a significant worsening for the following questions in the GCOM: “They tend to repeat something that someone just said” (*P=*.03); “They use filler words (‘thing,’ ‘whatchamacallit’) instead of precise words (‘pencil,’ ‘balloon’)” (*P=*.04).

Although the QoL questionnaire and the GCOM did not show a significant change, the semistructured interviews with all caregivers (n=17) revealed that they felt COMPAs had a positive impact on the lives of persons living with dementia (please refer to the *Qualitative Findings* section). Some caregivers described these positive impacts as positive emotions during nonverbal communication revealed by a positive facial expression in the person living with dementia or the simple fact that the person living with dementia started dancing while using COMPAs.

### Qualitative Findings

The qualitative analyses of semistructured interviews with the caregivers regarding the effects of COMPAs use with persons living with dementia in the LTC setting highlighted 3 main themes: capacity of COMPAs to elicit positive emotions, decrease in caregiver burden, and versatility of COMPAs.

#### Eliciting Positive Emotions

The interviews highlighted COMPAs’s capacity to elicit positive emotions in persons living with dementia and caregivers. This increase in positive emotions led to an increase in feelings of joy, pleasure, and happiness.

#### Persons With Dementia

Caregivers (16/16, 100%) reported on COMPAs’s capacity to elicit positive emotions in persons living with dementia, who had expressed well-being and the pleasure they got from their COMPAs session both verbally and nonverbally via changes in their facial expression (eg, smiles and eye contact):

This nonverbal person’s face would light up.

P6

Almost all caregivers discussed the positive effects of personalized content on persons living with dementia: how pictures or music from their past can evoke positive memories (15/17, 88%) and positive emotions (16/17, 94%). Persons living with dementia enjoyed reminiscing and sharing former moments of their life with their caregivers:

COMPAs calms them, and does them good too, because it reminds them of memories, good memories, there are pictures of their kids, their pet, their house.

P14

I would say cheerfulness. They were happy to see the pictures, listen to their favorite music.

P17

#### Caregivers

Caregivers (14/17, 82%) reported that using COMPAs gave them joy, pleasure, and overall positive effects on their daily lives. They said that they looked forward to using COMPAs with their patients and appreciated these moments in their week:

To me, it is my moment of pleasure, when I am working.

P5

It was good for them but for me too because I felt their well-being.

P17

Some of the caregivers (14/17, 82%) said that simply seeing the enjoyment of the persons living with dementia gave them pleasure too:

Yes, I would see that it brought them joy, so it brought me pleasure too.

P14

Caregivers also reported on how they appreciated the effects of the app on the persons living with dementia. They valued COMPAs’s capacity to contribute to the well-being of persons living with dementia:

It’s great, because we give the resident the chance not to feel lost, and without this tool, you can’t really do it.

P10

#### Decrease in Caregiver Burden

The analysis highlighted how COMPAs gave caregivers a solution to deal with their everyday struggles, helped them feel empowered, and resulted in better bonds with the residents.

COMPAs was used as a tool and, in some cases, as an excellent way out of difficult situations involving persons with dementia, such as opposition, disorientation, and apathy. Caregivers (12/17, 71%) saw COMPAs as a solution that worked with residents who had required more attention. They were grateful to have an effective solution in these types of cases:

Interviewer: Did you have the impression of having a solution?

P13: Yes, exactly, now I have a solution.

*P2:But, with some residents, the device also helps us to do the tasks.There are some cases that are more difficult, but with the device, it improves our interaction with certain residents a bit*.

The implementation of COMPAs also contributed to caregivers’ feelings of personal achievement in the workplace. Caregivers (15/17, 88%) felt more useful and believed that they made a real difference in the lives of the persons living with dementia, as they could go beyond providing primary care. Caregivers enjoyed learning about the residents, their lives, and their personal tastes, and they felt empowered by having an additional clinical role:

It lifts you up, in your work...you are not only there to help them with comfort care or to feed them.

P10

I can say it adds something good to the atmosphere, it adds more...How can I say this...I could say particularly with PWDs, it makes us want...with people who communicate less. It is like us; it makes us want to reach out to those people.

P6

Caregivers (11/17, 65%) discussed how COMPAs enabled the development of more personalized relationships with persons living with dementia. They felt closer to the residents and more interested in them. Spending more time with persons living with dementia helped caregivers to create a bond and spend quality time with them despite the communication difficulties:

P9: ...just spending fifteen minutes with them, it was a moment of joy and relaxation. Because I was also learning plenty of things.

Interviewer: Do you mean that it provided you with joy to spending time with them?

P9: Yes, exactly. We never take enough time; we do not take the time to talk to them.

Caregivers (11/17, 65%) also said that COMPAs allowed them to have better interactions with the residents. They talked about better-quality exchanges, and generally enhanced communication, including communication by the persons living with dementia themselves:

We can communicate with the device.

P14

Even their speech is more fluent.

P10

#### Versatility of COMPAs

Caregivers highlighted the flexibility of COMPAs, as it could be used for different reasons, in different settings, and for different durations.

COMPAs was implemented in different ways by different caregivers. They could adapt it to their working conditions, and over time, they incorporated the tool into their daily routine. Some caregivers used it while providing grooming care:

And there are moments, like grooming care, that are a bit stressful, I would put his music on, and we sang, we danced in front of the mirror, and we giggled.

P13

Some used it in a group setting, while others used it individually. Some caregivers had a fixed time in their day dedicated to COMPAs, while others used it at different times depending on the situation:

A big asset of COMPAs, is that in fact, we can use it at any time of day.

P11

The duration of a COMPAs session also varied between caregivers, ranging from 5 minutes to around 20 minutes:

When I have five minutes, ten minutes, I would take the iPad, go to the room, and we listened together. Sometimes when I have more time, l stay longer.

P4

#### Challenges for Caregivers

Caregivers reported some issues during the implementation of COMPAs. Lack of time, technological issues, and the responsibility for or availability of the device were mentioned as challenges.

#### Lack of Time

Close to half of the caregivers (8/17, 47%) stated that they lacked the time to use COMPAs. They commented that it was not always easy to take the time to conduct a COMPAs session because of workload or when time permitted, the resident might be unavailable:

The evening shift, it is hard to find the time to enjoy it.

P3

It was hard in the mornings with grooming care: there is too much work to do it properly. However, we would do, I would do one in the morning from time to time.

P15

#### Technological Issues

The participants encountered some technological issues during the implementation. Caregivers (3/17, 18%) discussed how the bugs could disrupt their sessions, making the residents lose interest in the content presented:

The videos did not work. I would have liked to do it with Mrs. B, watch videos, but it was not working.

P17

#### Responsibility for and Availability of the Device

Caregivers (6/17, 35%) raised the issue of being responsible for an iPad. Being responsible for a valuable object was a concern for them. In other cases, the iPad was locked, and a nurse had to make it available to the caregiver (5/17, 29%). Their busy schedules made it difficult for them to access the device when they needed it.

#### Challenges for Residents

A few residents (6/17, 35%) faced some problems while using COMPAs. Confusion, negative emotions, and disinterest were mentioned as challenges for residents.

Caregivers reported that some of their residents considered the app to be an intrusion; they did not understand how their personal information came to be in the attendant’s hand:

P10: People like Mrs. G, this dementia, well, for her, it’s not positive, because it’s difficult, she starts questioning. She wonders what is going on.

Interviewer: You think Mrs. G., it makes her wary?

P10: Mrs. G., she did it once and it was very hard to do it again, because she takes it as an intrusion.

Others were troubled by not being able to recognize the pictures shown to them. Negative emotions could also be elicited by the content:

A little bit of melancholy at times.

P12

Caregivers (4/17, 24%) reported that some residents were disengaged from COMPAs. This disinterest was related to the device, the redundancy of the content, or the resident’s attitude:

Well, there were some residents who weren’t even slightly interested.

P13

Some residents weren’t interested in watching the screen or were troubled by the screen.

P13

## Discussion

### Principal Findings

The purpose of this study was to implement and validate the effect of COMPAs, an app designed to elicit positive emotions triggering communication between persons living with dementia and their caregivers in an LTC setting.

Using a combination of quantitative and qualitative methods, the main results of the study validate our hypotheses. Specifically, the qualitative results from the semistructured interviews show that COMPAs improved person-centered communication between caregivers and persons living with dementia; its use resulted in more verbal and nonverbal exchanges in different contexts (eg, personal care and dedicated time). In particular, caregivers reported an improvement in the quality of exchanges and a more personal care relationship. Moreover, the use of COMPAs was associated with an improvement in QoL for both persons living with dementia and caregivers. The caregivers reported that COMPAs elicited positive emotions in persons living with dementia, contributing to emotional communication and helping the caregivers see the person living with dementia beyond the illness. In so doing, COMPAs supported person-centered care and communication between persons living with dementia and their caregivers. In addition, statistically significant results were observed in the form of increased caregiver empowerment, as reflected by the accomplishment score in the MBI. Caregivers also described COMPAs as a solution that helped them create opportunities to develop meaningful bonds with persons living with dementia, easing the caregiver communication burden. Finally, COMPAs was deemed well suited to the LTC context, particularly due to its versatility. These results were observed although 40% (7/17) of the caregivers were not accustomed to using an iPad, which provides evidence for the versatility of COMPAs in empowering even caregivers with limited technological literacy.

### Quality of Communication Between Persons Living With Dementia and Caregivers

The GCOM showed some deterioration in specific oral expression components for persons living with dementia, which is expected in the context of progressive conditions. Interestingly, the COMPAs intervention was associated with stable general communication skills in residents. This may be a result of the GCOM’s poor sensitivity to the communication patterns characterizing advanced neurocognitive disease, or it might reflect the benefits of daily stimulation with COMPAs in reducing morbidity, despite the progressive nature of neurocognitive disease [7]. The results on the GCOM also highlighted the positive nonverbal communication markers of well-being and positive emotions that COMPAs induced in residents, including smiling; raising eyebrows; touching the caregiver’s hand while coviewing; smiling, dancing, or singing to personalized music; and laughing with the caregiver. These findings highlight the app’s efficacy in promoting person-centered communication between LTC residents with dementia and their caregivers. They also attest to the benefits of COMPAs training, which increases caregivers’ awareness of nonverbal and emotional aspects of communication. These findings are in line with those of previous work showing that integrating communication strategies into care and using elements of a patient’s life story in informal discussions enhance meaningful communication between caregivers and LTC residents [6]. Moreover, the results of the semistructured interviews showed that following the COMPAs trial, caregivers focused more on nonverbal and emotional person-centered communication and less on verbal and transactional communication. These results reveal the importance of combining a good tool with suitable training in order to promote awareness of all dimensions of communication and the potential facilitators and barriers [31].

Studies conducted during the COVID-19 pandemic also found that COMPAs had positive effects on communication between caregivers and persons living with dementia in an LTC setting, even during periods of extreme isolation [47]. Specifically, caregivers reported that residents showed increased engagement, as opposed to apathy, together with verbal and nonverbal expressions of joy, well-being, and calmness while using COMPAs, despite major public health restrictions and the use of personal protective equipment [47].

### Residents’ and Caregivers’ QoL

#### Caregivers’ QoL

The results on the GHQ-12 showed that caregivers’ QoL increased following COMPAs use, and so did their sense of personal accomplishment (measured by the MBI-personal achievement scale). More specifically, an improvement in the feeling of personal accomplishment was observed in the level of energy that the caregiver felt when working closely with the resident. This is in line with the findings of previous studies showing that significant communication between persons living with dementia and caregivers is associated with a better QoL [60,61].

The analyses of semistructured interviews show that caregivers described COMPAs-supported interactions with residents as pleasant times, moments of relaxation, and even as therapeutic for them. Thus, caregivers saw COMPAs as a solution to their struggles with persons living with dementia: a quick and effective way out of the challenges they faced in managing difficult behaviors (eg, apathy and agitation) and engage in more natural communication. It was probably this factor that led to the association between reduced caregiver burden and COMPAs use. Similar findings were reported in previous work showing the relationship between caregiver burden and the quality of communication [9,62]. Furthermore, there was a decrease in the score of the MBI-depersonalization scale for over half of the participating caregivers (9/17, 53%). This may have been related to the personalized COMPAs content, which helps caregivers appreciate the person beyond the disease and become aware of the individual traits of the persons living with dementia including their history, culture, tastes, and preferences, that is, the opposite of depersonalization.

The caregivers expressed their satisfaction with knowing more about the person they were caring for, spending more time with them, getting to know their life story better, and seeing the residents happier in this context. Indeed, personalized content is shown to be relevant in facilitating communication among persons living with dementia [63]. Thus, improving the quality of communication had positive effects on the dyad and helped to establish an empathic relationship. As a result, caregivers felt valued and satisfied with their work:

It lifts you up in your job: you’re not there just to help them with their comfort care or feed them.

Using COMPAs empowered the caregivers, and this is probably a key reason for reduced caregivers’ burden.

#### Residents’ QoL

The QDV-DTA scores did not show any significant changes in the residents’ QoL. However, caregivers reported that COMPAs triggered positive expressions in the residents, demonstrated by their nonverbal communication. They viewed COMPAs as a tool helping residents change their routine, remember positive times, break out of their isolation, and feel well. They considered COMPAs to be a valuable tool to support meaningful communication, thus supporting social engagement in persons with dementia. During the semistructured interviews, caregivers mentioned COMPAs’s ability to support a meaningful activity, which meant persons living with dementia were involved in stimulating activities. In light of the literature, residents’ QoL is promoted by social contacts, a good relationship with the caregiver, and the caregiver’s involvement in providing care [26,28]. These factors are also associated with better self-esteem in persons living with dementia, an essential component of their well-being [5] and dignity [17]. Furthermore, it is essential to address the socialization needs of persons living with dementia and provide person-centered care [64]. In line with this literature and considering the verbal and nonverbal manifestations of well-being in residents documented by caregivers in the semistructured interviews, the results of this study prove the relevance of COMPAs for communication and QoL. They illustrate the value of orienting communication around emotional content linked to the residents’ own life trajectory, which improves interactions in the dyad, and in turn promotes positive relationships [16,33].

### Burden on Caregivers

QoL at work refers to various factors such as satisfaction, mental health, and stress level [65]; these 3 factors contribute to caregiver burden. More specifically, stress arises when caregivers fear not having the necessary resources to face the physical and psychological challenges that they may encounter in geriatric care. The reduction in caregiver burden and increased personal achievement found in this study may be related to several factors. One is the fact that caregivers received a training session on communication strategies and barriers in LTC. Thus, the literature shows that training on strategies for communicating with persons living with dementia improves caregivers’ communication skills and is associated with a decrease in their burden [61].

Another factor that may have contributed to reducing caregiver burden is reflected by the results on the MBI and by the thematic analyses concerning the stress and frustration associated with communication barriers [9], all of which were reduced in this project. Specifically, the quantitative results on the MBI following 8 weeks of COMPAs use showed a significant reduction in the burden score, concurrently with a significant improvement in the personal achievement score and a reduction in feelings of tiredness. Hence, COMPAs was a resource for caregivers, allowing them to feel more accomplished and less exhausted at work. These effects are also illustrated by the semistructured interviews; caregivers reported that adding COMPAs to their daily routine did not result in work overload.

In fact, caregivers noticed the positive effect of COMPAs on the residents and wanted to continue using the app, although they occasionally mentioned not having time to use COMPAs to their satisfaction or to add material to the libraries. The key will be to find more time in everyday life situations for caregivers to use COMPAs and to simplify the addition of personalized material. We are currently working on these 2 elements to meet these needs.

In sum, the results on the MBI and semistructured interviews show that COMPAs reduced caregiver burden, a factor that is associated with more relaxed care, which in turn further reduces their burden [66,67]. Furthermore, the emotional component of COMPAs interventions contributed to the expression of empathy, which is also known to reduce caregivers’ burden.

### Strengths and Limitations of the Study

To our knowledge, this is the first study to explore the implementation of a communication-based app in the context of LTC settings. The qualitative results of this study demonstrate COMPAs’s effectiveness in positively influencing the lives of persons living with dementia and caregivers in an efficient and timely manner. It should be noted that COMPAs’s positive effects on caregivers might perhaps be influenced by a selection bias; they volunteered for the study, and therefore, they might have a positive bias toward the method and would not necessarily be representative of the target population. However, according to the LTC administrators, the caregivers’ demographic profile in the sample was representative of the vast majority of caregivers in Québec and Canada, mostly immigrant women aged between 20 and 60 years. Following the launch of the project, more caregivers saw the benefits and told the research team that they wish they had enrolled. Future studies could explore COMPAs’s effects and adherence in a larger community of caregivers as a function of cultural background, age, gender, and technological literacy, among other things.

Finally, we acknowledge that the statistical significance of the quantitative results is limited. This may be a consequence of the small sample size and the diversity of residents’ clinical profiles. Hence, although we find medium effect sizes, these results should be considered as only a tendency. Future studies with larger samples of participants are required to confirm these results and test the generalizability of these findings in broader populations, including family caregivers of persons living with dementia in LTC residences or other LTC populations without dementia but with severe communication impairments following stroke, or severe sensory impairments in the context of behavioral disturbances, or major psychiatric disorders.

COMPAs interventions proved to be suitable for implementation in LTC residences, as caregivers could choose the duration, time, and modality of their sessions with persons living with dementia. Caregivers could adapt the app to their working conditions, making COMPAs a versatile tool that can be modulated to the users’ needs.

The literature shows that technology is underused to support the communication between caregivers and persons living with dementia in LTC settings [37], and no available evidence was found to support the use of apps for such communication. This study provides evidence that COMPAs is suitable to support person-centered care in the caregiver-LTC resident dyad. To our knowledge, this is the first app that supports communication through a person-centered care approach and that is fully customizable to the person living with dementia.

Although the information gathered from caregivers in the questionnaires and the semistructured interviews is an effective way to understand COMPAs’s effects on persons living with dementia, we acknowledge that the perspectives of persons living with dementia were not included. Including them in the research team entails ethical and logistical challenges [68,69]. To be in line with the person-centered care approach, future studies should include their point of view [32]. The research team created the personalized libraries, which saved time for the caregivers, who were overloaded with everyday work, but persons with dementia could be more involved in choosing the material. This will be done in future studies.

In a context where human resources and time are limited, it will be important to develop efficient ways to create user-friendly personalized libraries. Future work will focus on improving the COMPAs interface by adding artificial intelligence modules that will assist in more efficiently creating sophisticated personalized libraries.

### Conclusions

COMPAs proved to be effective in improving communication between caregivers and residents while reducing the burden on caregivers and improving both groups’ QoL.

The evidence shows that COMPAs facilitates person-centered communication. Positive emotions generated in residents resonate in caregivers, stimulating empathy and well-being in the dyad. This state of shared well-being promotes social engagement, defocuses attention from impairments and disabilities, and fosters exchanges between the dyad and what they share. The impressive gains in relevant outcome measures obtained with residents and caregivers underscore the relevance of COMPAs in LTC settings. Large-scale studies are necessary to validate the observed tendencies and optimize COMPAs’s potential benefits in persons with living dementia in LTC settings and their caregivers, while examining its use with other susceptible populations presenting communication deficits. Studies could also explore the barriers to technology use in caregivers and persons living with dementia and ways to overcome them; they must also consider the ethical issues related to technology use with susceptible populations, including privacy and security, to identify best practices for safe implementation of technology in dementia care.

## Abbreviations

BPSD: behavioral and psychological symptoms of dementia
COMPAs: Communication Proches Aidants
GCOM: grille d’évaluation des difficultés de communication dans la démence
GHQ-12: General Health Questionnaire-12
LTC: long-term care
MBI: Maslach Burnout Inventory
QDV-DTA: qualité de vie dans la démence de type Alzheimer
QoL: quality of life
RA: research assistant
SLP: speech language pathologist
